# The recombinant E^rns^ and truncated E2-based indirect enzyme-linked immunosorbent assays to distinguishably test specific antibodies against classical swine fever virus and bovine viral diarrhea virus

**DOI:** 10.1186/s12985-022-01851-w

**Published:** 2022-07-22

**Authors:** Weicheng Yi, Hongchang Zhu, Yihan Wu, Qingmei Li, Wange Lou, Haizhong Zhao, Zishu Pan

**Affiliations:** 1 State Key Laboratory of Virology, College of Life Sciences, Wuhan University, Wuhan, 430072 China; 2 Key Laboratory of Animal Immunology, Henan Academy of Agricultural Sciences, Zhengzhou, 450002 China; 3 Institute of Animal Husbandry and Veterinary Medicine, Hubei Academy of Agricultural Sciences, Wuhan, 430064 China

**Keywords:** Classical swine fever virus, Bovine viral diarrhea virus, E^rns^, E2, Serological diagnosis, Indirect enzyme-linked immunosorbent assay

## Abstract

**Background:**

Classical swine fever (CSF) virus is the causative agent of an economically important, highly contagious disease of pigs. CSFV is genetically and serologically related to bovine viral diarrhea virus (BVDV). BVDV infection in pigs can mimic CSF clinical signs, which cause difficulty in differentiation. Serological test for detection of virus specific antibodies is a valuable tool for diagnosis and surveillance of CSFV and BVDV infections in animals. The aim of this study was to develop the CSFV E^rns^ and BVDV tE2 -based ELISAs to distinguishably test specific antibodies against CSFV and BVDV.

**Methods:**

The CSFV E^rns^ and truncated E2 (tE2, residues 690–865) of BVDV were expressed in *E. coli* and purified by Ni–NTA affinity chromatography, respectively. Employing E^rns^ or tE2 protein as diagnostic antigen, indirect ELISAs were developed to distinguishably test specific antibodies against CSFV and BVDV. The specificity and sensitivity of ELISAs were evaluated using a panel of virus specific sera of pigs, immunized rabbits and immunized mice. A total 150 clinical serum samples from farm pigs were measured by the developed ELISAs and compared with virus neutralizing test (VNT).

**Results:**

Indirect ELISA was established based on recombinant CSFV E^rns^ or BVDV tE2 protein, respectively. No serological cross-reaction between antibodies against CSFV and BVDV was observed in sera of immunized rabbits, immunized mice or farm pigs by detections of the E^rns^ and tE2 -based ELISAs. Compared to VNT, the CSFV E^rns^ -based ELISA displayed a high sensitivity (93.3%), specificity (92.0%) and agreement rate (92.7%), and the sensitivity, specificity and agreement rate of BVDV tE2 -based ELISA was 92.3%, 95.2% and 94.7%, respectively.

**Conclusion:**

The newly developed ELISAs are highly specific and sensitive and would be valuable tools for serological diagnosis for CSFV and BVDV infections.

## Background

Classical swine fever (CSF) is a highly contagious disease of pigs, which is caused by classical swine fever virus (CSFV). CSFV belongs to the genus *Pestivirus* in the family *Flaviviridae*, along with bovine viral diarrhea virus (BVDV), border disease virus (BDV) and several newly found atypical pestiviruses [1, 2]. CSFV is a small enveloped virus with a single-stranded, 12.3 kb RNA genome of positive polarity. The genome is composed of one large open reading frame (ORF) flanked by two untranslated regions (5′UTR and 3′UTR) [3]. The ORF encodes a 3,898-amino-acid polyprotein, which is further processed into four structural proteins (C, E^rns^, E1, and E2) and eight nonstructural proteins (N^pro^, p7, NS2, NS3, NS4A, NS4B, NS5A, and NS5B) by virus-encoded and cellular proteases [4, 5]. Antibodies directed to E^rns^, E2 and NS3 have been demonstrated in infected animals [6–8]. E^rns^ and E2 antibodies displayed virus-neutralizing abilities and conferred protection against viral infection [9, 10].

BVDV causes economically important disease in cattle, and also infected pigs [11, 12]. BVDV infection in pigs may be presented with a great variability of clinical signs [13], leading to be clinically indistinguishable from CSF [11, 14]. Serological detection of virus-specific antibodies is extensively used for diagnosis and monitoring virus infection in animals. The virus neutralization test (VNT) is the gold standard test for the serological diagnosis of CSFV and BVDV infections and it is also used as the reference potency test for commercial vaccines. However, the requirement of cell culture, live virus, and a dedicated facility makes the test expensive and difficult to deploy. Alternatively, serological diagnosis by enzyme-linked immunosorbent assay (ELISA) is a valuable tool for surveillance of CSF in apparently disease-free area or for monitoring in a CSF eradication program. CSFV E^rns^ and E2 -based ELISAs have been developed for serological diagnosis of CSF and developed for the evaluation of CSF maker vaccines [8, 15–17]. However, ELISAs based on the full-length E2 protein of CSFV cannot discriminate anti-CSFV from anti-BVDV antibodies. So, a serological diagnostic assay with the potential to effectively distinguish CSFV from BVDV infection is urgently needed. In this study, we developed the indirect ELISAs based on the recombinant CSFV E^rns^ and truncated E2 (tE2) of BVDV to detect specific antibodies in swine sera, with a specificity and sensitivity comparable with VNT.

## Materials and methods

### Cells, viruses, animals and serum samples

Porcine kidney 15 (PK15) cells and Madin-Darby bovine kidney (MDBK) cells were obtained from the China Center for Type Culture Collection (CCTCC, Wuhan, China) and cultured in Dulbecco’s Modified Eagle’s Medium (DMEM) (Invitrogen, Carlsbad, CA, USA) supplemented with 10% fetal bovine serum (FBS) (Natocor, Cordoba, Argentina) in 5% CO_2_ at 37ºC. The CSFV Shimen strain (GenBank accession number AF092248.2) was generated from a cDNA clone (pSPT_I_/SM) [18] and propagated in PK15 cells. The BVDV I Hubei strain (GenBank accession number MZ484396) is a passaged variant of BVDV I NDAL strain (GenBank accession number AJ133738.1) [19] and propagated in MDBK cells. The BVDV Hubei strain caused typical cytopathogenic effect (CPE) similar to the parental BVDV NADL strain.

Twelve 5-week-old New Zealand White rabbits (Hubei Center for Disease Control and Prevention, China) were randomly divided into three groups (A to C), with four rabbits each. Rabbits in group A were intravenously (i.v.) injected with 1 ml of CSFV (10^6^ TCID_50_/rabbit). Rabbits in group B were i.v. injected with 1 ml of BVDV (10^6^ TCID_50_/rabbit). Rabbits in group C were i.v. injected with 1 ml DMEM as a control. All rabbits received a booster administration with the same dose at 2 week intervals. Blood samples were collected at day 7 after the last injection and stored at -80 °C for the subsequent assay.

Similarly, eighteen 6-week-old specific-pathogen-free (SPF) female BALB/c mice (Hubei Center for Disease Control and Prevention, China) were randomly divided into 3 groups (A to C), with six mice each. Mice in group A were intramuscularly (i.m.) injected with 100 μl of CSFV (10^5^ TCID_50_/mouse). Mice in group B were i.m. injected with 100 μl of BVDV (10^5^ TCID_50_/mouse). Mice in group C were i.m. injected with 100 μl DMEM as a control. All mice received a booster administration with the same dose at 2 week intervals. Blood samples were collected at day 7 after the last injection and stored at -80 °C for the subsequent assay.

One hundred and fifty swine serum samples were collected from pig farms in different regions of China, including Hubei, Henan and Guangxi Provinces. The twenty-four virus-specific sera, including PCV2-positive (n = 9), PRRSV-positive (n = 6), PEDV-positive (n = 4) or ASFV-positive (n = 5) sera, were collected from pig farms in Hubei and Henan Provinces.

### Cloning, expression and purification of recombinant E^rns^ and truncated E2

To construct the expression plasmids, the codon-optimized E^rns^ gene of CSFV Shimen strain and the coding sequence of truncated E2 (tE2, residues 690—865) of BVDV Hubei strain were synthetically produced (Sangon Biotech, Shanghai, China). The E^rns^ gene was amplified using the specific primers E^rns^-F (5′-CACCATGGCGGAAAATATCACCCAGTGGAACCTGAGCG-3′) (underline, *Nco*I site) and E^rns^-R (5′-CTCTCGAGCGCGTACGCGCCGAACCAGGTTTTGC-3′) (underline, *Xho*I site). The coding region of tE2 was amplified using the specific primers E2-F (5′-CACCATGGCACACCTGGATTGCAAACCGGAATTC-3′) and E2-R (5′-CTCTCGAGTTCACCCAGGTTTTTCTGGGTGATG-3′). The PCR products were cloned into expression vector pET-28a to generate the pET-E^rns^ and pET-tE2, respectively. Both resulting constructs were confirmed by sequencing.

Purification of recombinant E^rns^ or tE2 protein was performed as previously described [20, 21]. In brief, *E. coli* BL21-CodonPlus (DE3)-RIL was transformed with the plasmid pET-E^rns^ or pET-tE2, and then the bacteria were grown at 37 °C in LB medium containing 50 μg/ml kanamycin until the optical density at 600 nm (OD_600_) reached 0.6. Isopropyl-β-D-1-thiogalactopyranoside (IPTG) was then added to a final concentration of 0.5 mM, and the cells were grown for an additional 4 h at 25ºC. The culture cells were harvested and resuspended in the lysis buffer (300 mM NaCl, 50 mM NaH_2_PO_4_·2H_2_O, pH 8.0) for sonication. After centrifugation, the pellets were collected, washed, and then resuspended in the binding buffer (300 mM NaCl, 50 mM NaH2PO_4_, 8 M Urea, 5 mM Imidazole, pH 8.0) and the targeted protein was purified using His Trap HP column (GE Healthcare, Freiburg, Germany) according to the manufacturer’s protocol. The purified protein was quantified using the Bradford assay kit (Sangon Biotech) and stored at -80ºC for subsequent experiments.

### SDS-PAGE and western blotting

The harvested samples were subjected to 12% SDS-PAGE and subsequently characterized by Western blotting as described previously [22]. In briefly, the protein samples were separated on 12% polyacrylamide gels and stained with Coomassie blue R-250 or transferred onto nitrocellulose membranes for Western blot analysis with a mouse anti-His monoclonal antibody (ABclonal, Wuhan, China) and rabbit anti-CSFV or rabbit anti-BVDV polyclonal antibody (prepared in our laboratory) as primary antibody (1:10,000 dilution), followed by incubation with horseradish peroxidase (HRP)-conjugated goat anti-mouse/anti-rabbit IgG (ABclonal).

### Establishment of the E^rns^ and tE2 -based indirect ELISAs

The optimal concentrations of the coating antigen, dilutions of sera and the secondary antibody [HRP-conjugated rabbit anti-pig IgG (Sigma-Aldrich, St. Louis, MO, USA)] were determined according to the checkerboard titration method [23]. Briefly, 96-well ELISA plates (BIOFIL, Guangzhou, China) were separately coated with the purified antigen at concentration of 2.5, 5, 10 or 20 μg/ml in coating buffer (pH 9.6) at 4ºC overnight. After washing with PBST (PBS containing 0.05% Tween-20) and blocking with 3% bovine serum albumin (BSA) (Biosharp, Hefei, Anhui, China), the diluted swine sera were added to coated plates (100 μl/well), respectively, and incubated at 37ºC for 1 h. After washing with PBST, the diluted secondary antibodies were added to the plates (100 μl/well) for 1-h incubation at 37ºC. After washing with PBST, the bound antibodies were detected with tetramethylbenzidine (TMB) substrate (100 μl/well) at room temperature. The reaction was stopped with 2 M H_2_SO_4_ (100 μl/well). The optical density (OD) values were measured at 450 nm using a microplate reader (Thermo Scientific, Waltham, MA, USA). The optimal conditions were determined according to the positive control/negative control (P/N) values in absorbance at OD450nm.

### Evaluation of the specificity and sensitivity of the E^rns^ and tE2 -based indirect ELISAs

Specificity and sensitivity of the E^rns^ and tE2 -based ELISAs were tested using150 swine sera. Specific antibodies directed to CSFV and BVDV in these sera were detected by virus neutralization test (VNT). The cut-off values of the E^rns^ and tE2 -based ELISAs were determined by SPSS software version 22.0 (https://www.ibm.com/analytics/spss-statistics-software). The sera from pigs clinically infected other viruses, including PCV2, PRRSV, PEDV and ASFV were used to evaluate cross-reactivity within the assay. The specificity of the E^rns^ and tE2 -based ELISAs was confirmed using specific anti-CSFV and anti-BVDV sera of immunized mice and immunized rabbits. The sensitivity of the assays was evaluated using 4 serum samples with different neutralizing antibody (NAb) titers.

### Virus neutralization test

Virus neutralization test (VNT) was performed as described previously [24, 25]. Briefly, serum samples were heat-inactivated for 30 min at 56ºC. Replicates of two-fold serially diluted sera (50 μl/well, starting from 1/4) were mixed with an equal volume of 100 TCID_50_ of CSFV or BVDV and incubated at 37 °C for 1 h. The mixture was then transferred to the PK15 (for CSFV) or MDBK (for BVDV) cell monolayers in 96-well plates and incubated at 37 °C in 5% CO_2_ atmosphere. For CSFV neutralization, after 72 h of incubation the cell monolayers were fixed in cold 50% (v/v) methanol/acetone for 30 min at -20 °C and subjected to immunofluorescence staining with rabbit anti-NS3 polyclonal antibody [26] and Alexa Fluor 488 -conjugated goat anti-rabbit IgG (ABclonal). NAb titer was expressed as the reciprocal of the highest dilution that caused complete neutralization. For BVDV neutralization, after 96 h of inoculation the CPE of MDBK cells in culture plates was observed under microscopy. NAb titer was read as the highest dilution of serum resulting in 50% CPE reduction. Sera with neutralizing titers > 1/16 were considered positive.

### Detection of clinical serum samples

Specific antibodies against CSFV and BVDV in 150 swine serum samples were measured by the developed E^rns^ or tE2 -based ELISA, respectively. The swine serum samples diluted with PBST containing 1% BSA (1:200) were used to ELISA tests. The positive and negative sera tested by the ELISA assays were compared with VNT.

## Results

### Expression and purification of recombinant CSFV E^rns^ and BVDV tE2 proteins

**Fig. 1:**
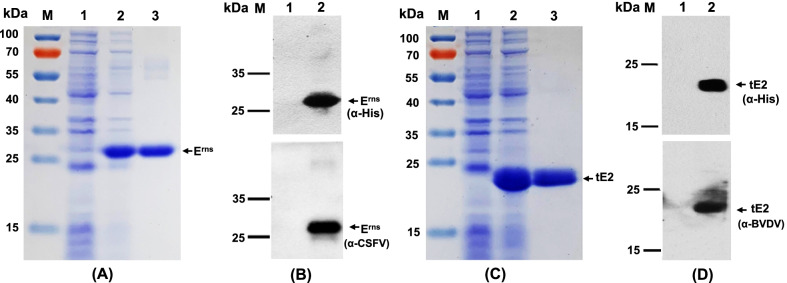
Expression and identification of CSFV E^rns^ and BVDV tE2 proteins. **A** & **C** The recombinant CSFV E^rns^ (**A**) or BVDV tE2 (**C**) protein expressed in *E. Coli* was analyzed by SDS-PAGE, with Coomassie blue staining. M, molecular marker; Lane 1, lysate of bacteria harboring the control plasmid pET-28a; Lane 2, the lysate of bacteria harboring the plasmid pET-E^rns^ (**A**) or pET-tE2 (**C**); Lane 3, the purified CSFV E^rns^ (**A**) or BVDV tE2 (**C**) protein. **B** & **D** Recombinant CSFV E^rns^ (**B**) or BVDV tE2 (**D**) protein was confirmed by western blotting using an anti-His monoclonal antibody (upper) or a specific anti-virus polyclonal antibody (lower). M, molecular marker; Lane 1, lysate of bacteria harboring the control plasmid pET-28a; Lane 2, the lysate of bacteria harboring the plasmid pET-E^rns^ (**B**) or pET-tE2 (**D**)

The recombinant CSFV E^rns^ or BVDV tE2 protein (fused with His-tag) expressed in *E. coli* was analyzed by SDS-PAGE (Fig. 1A, C), and confirmed by Western blot analysis using anti-His monoclonal antibody, rabbit anti-CSFV or anti-BVDV polyclonal antibody (Fig. 1B, D), respectively. Data showed that the targeted protein of approximately 26.7 kDa or 21.2 kDa was detected in *E. coli* lysates but not in negative control samples (Fig. 1). Western blot analysis identified that recombinant CSFV E^rns^ and BVDV tE2 proteins expressed in *E. coli* displayed specific reactivity with the corresponding anti-CSFV and anti-BVDV antibodies, respectively (Fig. 1B, D). The cell lysates containing the targeted proteins were purified with Ni–NTA affinity chromatography, and highly pure CSFV E^rns^ and BVDV tE2 proteins were prepared (Fig. 1A, C).

### Optimization of the CSFV E^rns^ and BVDV tE2 -based indirect ELISAs

**Fig. 2:**
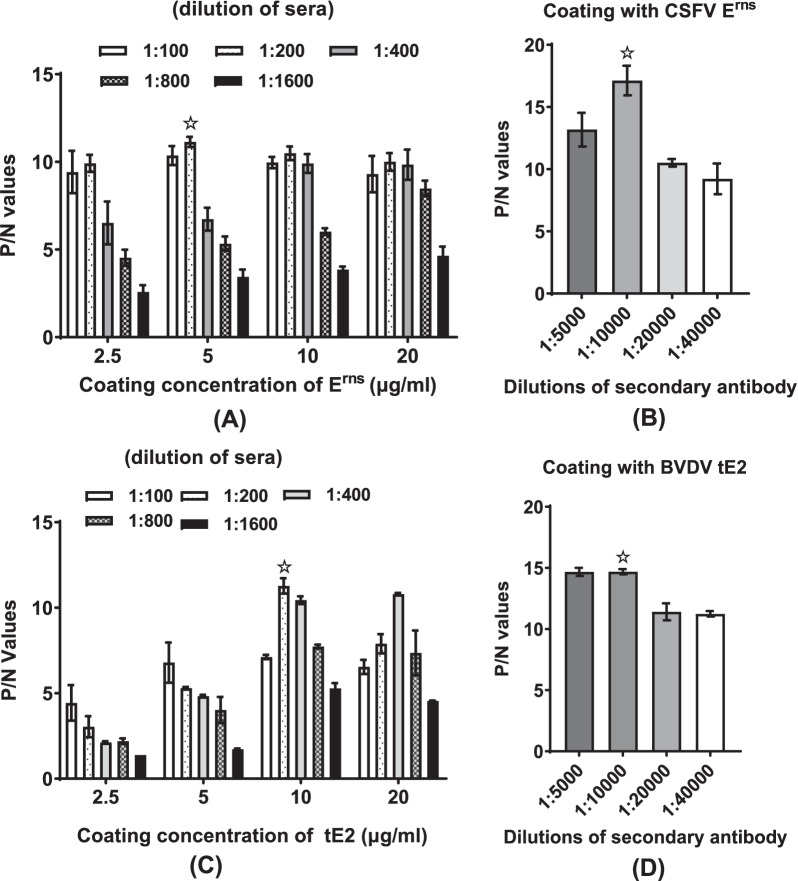
Optimizations of CSFV E^rns^ and BVDV tE2 -based ELISA procedures. **A** & **C** Optimization of the concentration of coating antigen and the dilution of swine sera for CSFV E^rns^ (**A**) or BVDV tE2 (**C**) -based ELISA using checkerboard titration test. **B** & **D** Optimization of the dilution of the secondary antibody for CSFV E^rns^ (**B**) or BVDV tE2 (**D**) -based ELISA. P/N, positive control/negative control; ☆, the optimized condition for ELISA test

**Fig. 3:**
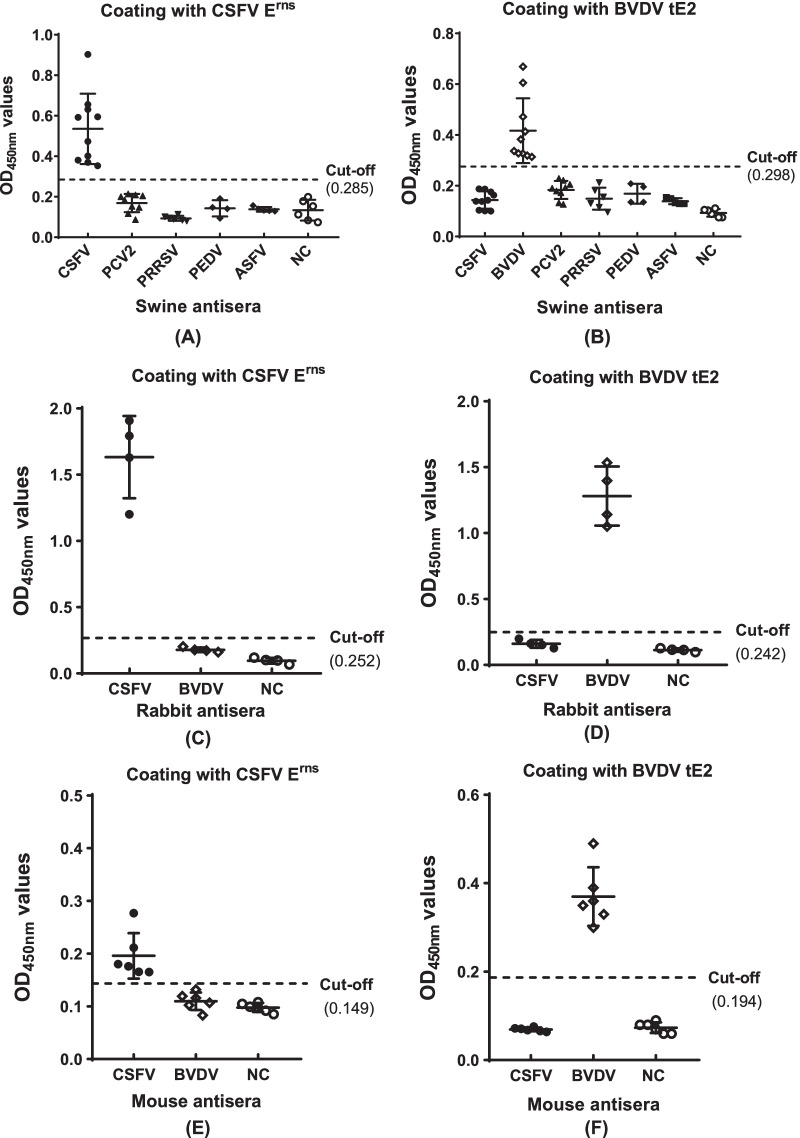
Validation of the specificity of CSFV E^rns^ and BVDV tE2 -based indirect ELISAs. The specificities of CSFV E^rns^ -based (**A**, **C** & **E**) and BVDV tE2 -based ELISAs (**B**, **D** & **F**) were validated using a panel of infected swine sera (**A** & **B**), including PCV2 (n = 9), PRRSV (n = 6), PEDV (n = 4), ASFV (n = 5), CSFV (n = 10), BVDV (n = 10) and negative control (n = 6); a panel of immunized rabbit sera (C & D), including CSFV (n = 4), BVDV (n = 4) and negative control (n = 4) or immunized mouse sera (**E** & **F**), including CSFV (n = 6), BVDV (n = 6) and negative control (n = 6)

Data of checkerboard titration showed that the optimized parameters of CSFV E^rns^ -based ELISA were the concentration of the coating antigen as 5 μg/ml, dilution of swine serum samples as 1:200 and dilution of secondary antibody dilution as 1:10,000 (Fig. 2A). Similarly, the optimized parameters of the BVDV tE2 -based ELISA were the concentration of the coating antigen as 10 μg/ml, swine serum samples as 1:200 dilution and secondary antibody dilution as 1:10,000 (Fig. 2B). Employing the optimal conditions, a total of 150 swine serum samples were tested using the CSFV E^rns^ and BVDV tE2 -based indirect ELISAs. The cut-off was calculated by receiver operator characteristic (ROC) analysis [8] for maximum diagnostic sensitivity and specificity using SPSS software. The cut-off absorbance values were set at 0.285 and 0.298, respectively (Fig. 3A, B). Samples with OD_450nm_ values greater than or equal to the cut-off value were considered positive.

### Specificity and sensitivity of the developed ELISAs

The specific anti-CSFV and anti-BVDV sera of pigs were used to determine the specificity of the CSFV E^rns^ and BVDV tE2 -based indirect ELISAs, and CSFV- and BVDV-free sera of pigs were used as negative control. Other virus-specific sera from the pigs clinically infected with PCV2, PRRSV, PEDV and ASFV were used to evaluate cross-reactivity within the assay. As expected, the absorbance values of all CSFV- and BVDV-free sera were lower than the defined cut-off value, and no cross-reactivity was detected by the coating antigen ELISAs between specific anti-sera directed to CSFV and BVDV. In addition, the absorbance values of all other virus-specific sera of pigs exhibited lower than the cut-off value by the CSFV E^rns^ and BVDV tE2 -based ELISAs (Fig. 3A, B). Because no specific BVDV antibody-positive sera (CSFV antibody-negative) of pigs were collected, the specificity of the assays was further confirmed using specific anti-CSFV and anti-BVDV sera of immunized rabbits and mice. Data showed that no cross-reactivity of the assays were observed between specific CSFV and BVDV antisera (Fig. 3C–F), which further confirmed that the coating antigen CSFV E^rns^ and BVDV tE2 possessed good specificity.

**Fig. 4:**
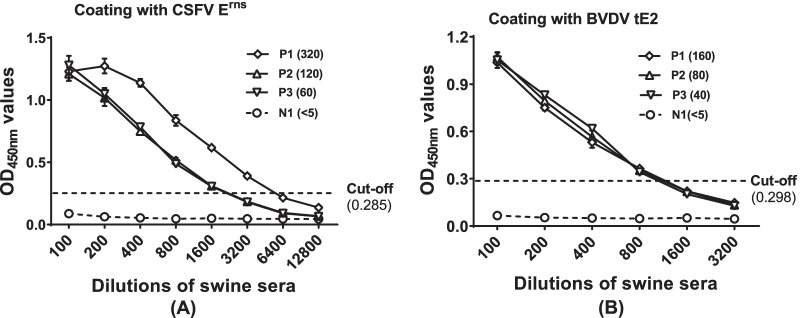
The sensitivity of CSFV E^rns^ and BVDV tE2 -based ELISAs. **A** Evaluation of the sensitivity of CSFV E^rns^ -based ELISA using serially swine sera with different neutralizing antibody (NAb) titers. P1, P2 and P3, anti-CSFV positive sera; N1, CSFV-free serum. The number in brackets indicated the NAb titer. **B** Evaluation of the sensitivity of BVDV tE2 -based ELISA using serially swine sera with different NAb titers. P1, P2 and P3, anti-BVDV positive sera; N1, BVDV-free negative serum. The number in brackets indicated the NAb titer

The sensitivity of the CSFV E^rns^ and BVDV tE2 -based ELISAs were evaluated using specific CSFV and BVDV sera with different NAb titers. Data showed that the ELISA assays displayed higher sensitivity for both specific anti-CSFV and anti-BVDV serum samples compared to the corresponding NAb titers (Fig. 4A, B).

### Detection of clinical sera samples

A total of 150 swine serum samples were used to evaluate the potential of the CSFV E^rns^ and BVDV tE2 -based indirect ELISAs. Compared with VNT, the CSFV E^rns^ -based ELISA displayed a high sensitivity (93.3%, 70/75) and specificity (92.0%, 69/75) with 92.7% agreement rate (139/150) (Table 1). Similarly, the sensitivity, specificity and agreement rate of BVDV tE2 -based ELISA was 92.3%, (24/26), 95.2% (118/124) and 94.7% (142/150) comparable to VNT, respectively (Table 2).

**Table 1:** Comparison of the CSFV E^rns^ -based indirect ELISA with virus neutralization test for detection of clinical serum samples of pigs

	Virus neutralization test		
	+	–	Total
ELISA			
+	70	6	76
–	5	69	74
Total	75	75	150
Agreement	93.3% (70/75)	92.0% (69/75)	92.7% (139/150)

+ , positive; –, negative

**Table 2:** Comparison of the BVDV tE2 -based indirect ELISA with virus neutralization test for detection of clinical serum samples of pigs

	Virus neutralization test		
	+	–	Total
ELISA			
+	24	6	30
–	2	118	120
Total	26	124	150
Agreement	92.3% (24/26)	95.2% (118/124)	94.7% (142/150)

+ , positive; –, negative

## Discussion

CSF makes a severe social and economic impact on global pig industry [27, 28]. Infection caused by BVDV in pigs has been reported in many countries [11, 29]. BVDV infection in pigs can mimic many of CSF clinical signs, leading to CSFV diagnosis and prevention problems [29, 30]. Rapid and accurate serological diagnosis is particularly crucial for evaluation of vaccination effects and surveillance of CSF. VNT is the gold standard for detection of CSFV specific antibodies, but detection of neutralizing antibody is time consuming, needs skill and well setup cell culture laboratory.

Among the structural and nonstructural proteins of CSFV, the E^rns^, E2 and NS3 can induce detectable antibodies [26, 31–33]. Many indirect ELISAs based on recombinant E2, E^rns^ or NS3 are available for specific antibody detection [8, 20, 31, 34, 35]. Because E2 is the primary target for neutralizing antibody [9, 36], the E2 -based ELISAs are widely used for CSF diagnosis and evaluation of vaccination efficiency. However, the available E2 -based ELISA tests can not distinguish CSFV from BVDV infection and usually lead to false positive [34, 37]. Serological cross-reactivity between specific antibodies against CSFV and BVDV in pigs [29, 38] is one of the obstacles for accurate diagnosis and CSF eradication program. Analysis of the structure showed that the amino acid residues 690–865 in *pestivirus* E2 form the important antigenic regions (the domains I/II or DA/DB), and the conserved amino acid sequences of these domains from BVDV and CSFV E2 are not identical (El Omari et al., 2013; Li et al., 2013b). Currently, an indirect ELISA for specific detection of antibodies against CSFV based on the truncated E2 of CSFV (containing amino acids 690—879 in E2) expressed in mammalian cells has been reported, which did not cross-react serologically with anti-BVDV sera [17]. Previous reports demonstrated that the CSFV and BVDV proteins prepared from both prokaryotic and eukaryotic expression systems were used as the antigens for diagnostic ELISAs [8, 17, 34, 39]. Although the biological activities of E^rns^ and E2 glycoproteins expressed in *E. coli* may not be justifiable in the absence of post-translational modification, its use in the diagnostics may have the advantage of being economic, productive and fast.

In this study, we prepared the CSFV E^rns^ protein and the E2 antigenic region (containing residues 690–865) of BVDV using a bacterial expression system and following Ni–NTA affinity purification. We developed the CSFV E^rns^ -based ELISA for specific detection of antibodies against CSFV and BVDV tE2 -based ELISA for specifically detecting anti-BVDV sera, respectively. Analyses of specificity and sensitivity of assays demonstrated that the E^rns^ -based ELISA was highly specific for CSFV antibody detection and that tE2 -based ELISA could specifically recognize BVDV antibodies. No serological cross-reactions between anti-CSFV and anti-BVDV sera were observed from specific virus protein-based ELISAs (Fig. 3). Compared to specific CSFV or BVDV test with different NAb titers, recombinant virus protein-based ELISA exhibited similar or higher sensitivity. The close correlation was obtained between NAb titers and indirect ELISA titers (Fig. 4).

We tested specific CSFV and BVDV antibodies of 150 clinical serum samples from pigs using the developed E^rns^ and tE2 -based ELISAs and the specific antibodies in these serum samples were further confirmed using VNT. Our data showed that the CSFV E^rns^ and BVDV tE2 -based ELISAs were sensitive and specific assays to distinguishably test CSFV and BVDV antibodies in sera of pigs. In 150 clinical samples, twenty-six sera exhibited both positive anti-CSFV and anti-BVDV antibodies, suggesting that infection of BVDV or co-infection of CSFV and BVDV existed in vaccination status of pigs involving large herd.

The systematic use of marker vaccines and stamping out have been the most successful tools for the control and elimination of the disease [40–42]. Chimeric CSFV containing BVDV E2 protect pigs against lethal CSFV challenge and induce a distinguishable antibody response [43]. Similarly, we currently constructed and characterized the chimeric rCSFV/bE2 with a substitution of BVDV E2 [19]. The sera of rabbits vaccinated with rCSFV/bE2 and CSFV vaccine C strain were tested using the developed CSFV E^rns^ and BVDV tE2 -based ELISAs, respectively. The antisera of rabbits injected with rCSFV/bE2 were both positive detection for specific anti-E^rns^ and anti-tE2, but antisera of rabbits injected with CSFV vaccine C strain were only positive detection for specific anti-E^rns^ (data not shown). Therefore, detection of the specific antibodies against CSFV E^rns^ and BVDV E2 could be an appropriate CSFV-specific marker ELISA test. Taken together, the CSFV E^rns^ and BVDV tE2 -based ELISAs established in this study were highly specific and sensitive and have the potential for serological surveillance of CSFV and BVDV infections and for serological differentiation of CSFV marker vaccination from wild type CSFV infection.

## Conclusions

In summary, in the present study, the CSFV E^rns^ and the truncated E2 (residues 690–865) proteins of BVDV were expressed in *E. coli*. and purified by Ni–NTA affinity chromatography, respectively. Using the E^rns^ or tE2 as diagnostic antigen, we developed indirect ELISAs to distinguishably test specific antibodies against CSFV and BVDV. The E^rns^ and tE2 -based ELISAs displayed a high sensitivity, specificity and the agreement rate relative to virus neutralizing test. No serological cross-reaction between two ELISA detections was observed in sera of immunized rabbits, immunized mice or farm pigs. The newly established ELISAs are valuable tools for diagnosis and surveillance of CSFV and BVDV infections and for serological differentiation of CSFV marker vaccination from CSFV natural infection.

## Data Availability

The data that support the findings of this study are available from the corresponding author upon reasonable request.
